# Hypoalbuminemia as predictor of recurrence of *Clostridium difficile* infection

**DOI:** 10.1007/s00508-018-1432-y

**Published:** 2019-01-07

**Authors:** Daniela Knafl, Matthias G. Vossen, Christian Gerges, Elisabeth Lobmeyr, Mario Karolyi, Ludwig Wagner, Florian Thalhammer

**Affiliations:** 1 Division of Nephrology and Dialysis, Department of Medicine III, Medical University of Vienna, Währinger Gürtel 18–20, 1090 Vienna, Austria; 2 Division of Infectious Diseases and Tropical Medicine, Department of Medicine I, Medical University of Vienna, Währinger Gürtel 18–20, 1090 Vienna, Austria; 3 Division of Cardiology, Department of Medicine II, Medical University of Vienna, Währinger Gürtel 18–20, 1090 Vienna, Austria; 4 Department of Emergency Medicine, Medical University of Vienna, Währinger Gürtel 18–20, 1090 Vienna, Austria; 5 4th Medical Department of Infectious Diseases and Tropical Medicine, Kaiser-Franz-Josef Hospital, Kundratstraße 3, 1100 Vienna, Austria

**Keywords:** Clostridium difficile, ATLAS, Serum albumin, Recurrence, Mortality, Antibiotic associated diarrhoea

## Abstract

**Background:**

Novel drugs for *Clostridium difficile* (*C. difficile*) infections have been proven to reduce recurrent infections. Because of their high financial costs, identification of patients at high risk for recurrence is essential to provide optimal treatment. The ATLAS score’s ability to predict 90-day recurrence, disease complications and 1‑year all-cause mortality was evaluated.

**Methods:**

144 consecutive symptomatic patients with positive stool test for *C. difficile* were enrolled. The ATLAS score (consisting of the variables age, temperature, leukocyte count, albumin, systemic antibiotics, serum creatinine) was calculated and patients were stratified into 4 subgroups according to their scores. A Cox regression model was used to estimate the extent to which ATLAS was associated with 90-day recurrence. Furthermore, the score was correlated with disease complications and one-year all-cause mortality.

**Results:**

ATLAS was unable to predict 90-day recurrence (*p* = 0.064, HR 1.134 [0.993;1.295]), but performed well for disease complications (*D* = 0.382, *p* < 0.001, HR 1.547 [1.266;1.889]) and mortality (*p* < 0.001, HR 1.374 [1.194;1.583]). Serum albumin was the only parameter able to predict 90-day recurrence (*p* = 0.016, HR 0.958 [0.926;0.992]) and was also a predictor of disease complications (*p* < 0.001, HR 0.865[0.809;0.924]) and one-year all-cause mortality (*p* < 0.001, HR 0.923 [0.896;0.950]). A threshold of 33.1g/L (sensitivity = 56%, specificity = 80%, AUC 0.683) and 29.2g/L (sensitivity = 75%, specificity = 70%, AUC 0.763) of serum albumin could be identified to be predictive for 90-day recurrence and one-year all-cause mortality, respectively.

**Conclusions:**

Serum albumin and ATLAS are predictors of disease complications and mortality, while only serum albumin is significantly associated with 90-day disease recurrence.

## Introduction

*Clostridium difficile* (*C. difficile*) infections (CDI) are the most frequent cause of healthcare-associated diarrhea, complicating the course of disease and prolonging hospitalization. Disease complications range from an asymptomatic carrier state to life-threatening infections [1, 2]. Fatal outcomes have been primarily attributed to recurrent *C. difficile*-associated diarrhea [3, 4]. Furthermore, the vicious circle of repetitive episodes of diarrhea leads to dehydration, intestinal protein loss, hypoalbuminemia, exhaustion and can eventually lead to death [5].

Novel drugs, such as fidaxomicin, a narrow spectrum macrocyclic antibiotic, and bezlotoxumab, a fully human monoclonal antibody that binds and neutralizes *C. difficile* toxin B, have been shown to be associated with a significant lower rate of recurrent infection [3, 6–13]. Despite contradictory findings derived from computer-based models that bezlotoxumab is cost-effective in the prevention of recurrent CDI compared with placebo, these drugs are expensive and may not be suitable as first-line treatment for CDI, which has been shown by economic analyses [14–16]. It may be suggested that these drugs should rather be reserved for a selected group of patients, which are at high risk for CDI recurrence [17].

Efforts have been made to discover a prognostic parameter for the early identification of patients with an increased risk for recurrence of CDI [10, 18, 19]. As a consequence early targeted treatment of high-risk patients with expensive therapies would be possible and financially feasible [17].

In the two global phase 3 trials MODIFY I and MODIFY II, bezlotoxumab demonstrated a statistically significant reduction in CDI recurrence, compared to placebo [10]. Gerding et al. showed that the risk factors specified in the MODIFY statistical analysis plan (age ≥65 years, history of CDI, compromised immunity, severe CDI, ribotype 027/078/244) were appropriate to identify patients at high risk for recurrence [12].

In order to find one simple parameter for the prediction of recurrent CDI, this study evaluated the ATLAS score and its single components. The ATLAS score is a bedside severity scoring system (Table 1), which has been shown to predict response to therapy, probability of colectomy and mortality but failed to predict CDI recurrence [20–25]. This study evaluated the potential association of ATLAS and its single components at onset of diarrhea with 90-day recurrence, 1‑year all-cause mortality and disease complications.

**Table 1:** Parameters of the ATLAS score [20]

Parameter	0 points	1 point	2 points
Age (years)	<60	60–79	≥80
Temperature (°C)	≤37.5	37.6–38.5	≥38.6
Leukocyte count (cells/μl)	<16,000	16,000–25,000	>25,000
Albumin (g/l)	>35	26–35	≤25
Systemic antibiotics^a^	No	–	Yes
Serum creatinine (μmol/l)	≤120	121–179	≥180

*CDI* Clostridium difficile infection

^a^During *Clostridium difficile* infection treatment (≥1 day)

## Material and methods

### Study design

A total of 150 consecutive, adult patients with a first episode of *C. difficile* infections, who presented at the University Hospital of the Medical University of Vienna, were enrolled between 1 January, 2012 and 31 December 2012. Diagnosis was established in patients with diarrhea (>3 stools per day, Bristol stool score 6 or 7) and a laboratory confirmed positive test for *C. difficile* with at least one of the following methods: a positive chemiluminescent immunoassay for *C. difficile* toxin and antigen from stool (LIAISON® GDH Assay, DiaSorin, Saluggia, Italy), a positive stool PCR for toxicogenic *C. difficile* strains (GeneXpert®, Cepheid, Sunnyvale, CA, USA). Patients with a history of *C. difficile* infection and under immunosuppressant treatment were excluded from the study. Furthermore, patients who received treatment for CDI, which have shown to reduce the risk of recurrence (e. g. fidaxomicin), were excluded from analyses.

The 90-day recurrence, severity, and 1‑year all-cause mortality served as main outcomes. The 90-day recurrence was defined as diarrhea with a laboratory confirmed positive stool test for *C. difficile* after at least one stool sample was tested negative within the first 90 days after the first episode of CDI. Disease complications were stratified into: (1) no complications, (2) mild complications (AE), (3) severe complications (SAE). Mild complications included unfavorable symptoms, such as meteorism, abdominal pain or need for pain medication, while life-threatening conditions, need of treatment in an intensive care unit (ICU), prolongation of hospitalization, disease resulting in persistent or significant disability, the need of surgical intervention, the occurrence of toxic megacolon, ileus, shock or death were defined as severe complications. The 1‑year all-cause mortality was defined as death of any cause 1 year after laboratory diagnosis of CDI was established.

The Ethics Committee (EC) of the Medical University of Vienna approved the conduct of the study and all patients signed informed consents (EC number 1898/2014).

### Statistical analysis

Normality was assessed by the Kolmogorov-Smirnov test. Normally distributed data were described as means ± standard deviations. Qualitative variables were described with counts and percentages. The potential of the ATLAS score and its single variables (age, temperature, leukocyte count, serum albumin, systemic antibiotic treatment and serum creatinine) to predict 90-day recurrence and survival was tested using univariate Cox regression analyses. Furthermore, the potential of serum albumin to predict 90-day recurrence and mortality was assessed with receiver operating characteristic (ROC) curves. Cut-off values were determined by maximizing the Youden index, which is the sum of sensitivity and specificity −1. Furthermore, Kaplan-Meier estimates stratified by ATLAS and albumin groups were utilized to assess survival. The logrank test was used to compare the survival distributions between the groups. Univariate logistic regression analyses were performed in order to assess the association between the ATLAS score and its single variables and disease complications. In addition, the coefficient of association Somers’ D was calculated due to the ordinal nature of the ATLAS score. Data were analyzed with SPSS Statistics (Version 21 for Mac, IBM Cooperation, Armonk, NY, USA). All *p*-values result from two-sided tests, with significance inferred at *p* < 0.05.

## Results

Of the 150 included patients, 144 met the prespecified inclusion criteria, 1 patient had to be excluded due to insufficient clinical data, 2 patients were excluded because *C. difficile *was not microbiologically confirmed and a further 3 patients were excluded because they received fidaxomicin as primary therapy for CDI. Age ranged between 19 and 96 years and 79 (54.9%) were female. The ATLAS group 5–7 was most abundant (38.9%; Table 2).

**Table 2:** Demographics and laboratory values

Characteristics	All patients (*n* = 147)
*Sex, no. (%)*	
Female	79 (54.9)
*Age, in years no. (%)*	
≤60	58 (39.5)
60–79	59 (41.0)
≥80	27 (18.8)
Mean ± SD	60.5 ± 20.4
*Temperature in °C, no. (%)*	
≤37.6	91 (63.2)
37.7–38.5	30 (20.8)
≥38.6	23 (16.0)
*Leukocyte count in G/l, no. (%)*	
<16	123 (85.4)
16–25	13 (9.0)
>25	8 (5.6)
*Serum albumin in g/l, no. (%)*	
>35	48 (33.3)
26–35	67 (46.5)
<25	29 (20.1)
*Serum creatinine in μmol/l, no. (%)*	
<120	93 (64.6)
121–179	25 (17.4)
>180	26 (18.1)
*ATLAS subgroups*	
Group 1–2	36 (25.0)
Group 3–4	45 (31.3)
Group 5–7	56 (38.9)
Group 8–12	7 (4.9)

**Table 3:** 90-day recurrence rate, 1‑year all-cause mortality, and disease complications

Characteristics	All patients (*n* = 144)
*90-day recurrence, no (%)* ^*a*^	45 (34.1)
*1-year all-cause mortality, no (%)* ^*b*^	44 (30.6)
*Complications, no (%)*	
*No complications (1)*	71 (49.3)
*Mild complications (2)*	24 (16.7)
*Severe complications (3)*	49 (34.0)

^a^Available from 132 patients

^b^Available from 133 patients

Of the patients 76 received metronidazole as first-line therapy, of which 68 received the drug orally and 8 intravenously, 1 patient received oral teicoplanin, 7 patients received oral vancomycin, and 2 received rifaximin. Of the patients 13 received symptomatic treatment only. In 45 patients the chosen antimicrobial therapy was not available; however, none of the patients received bezlotoxumab, as this drug was not available during the study period at the study location. Data of 132 patients regarding 90-day recurrence were available of which 45 patients (34.1%) experienced recurrence of disease (Table 3). The 90-day recurrence of *C. difficile* diarrhea was highly associated with increased 1‑year all-cause mortality (*p* < 0.001).

### 90-day recurrence

**Fig. 1:**
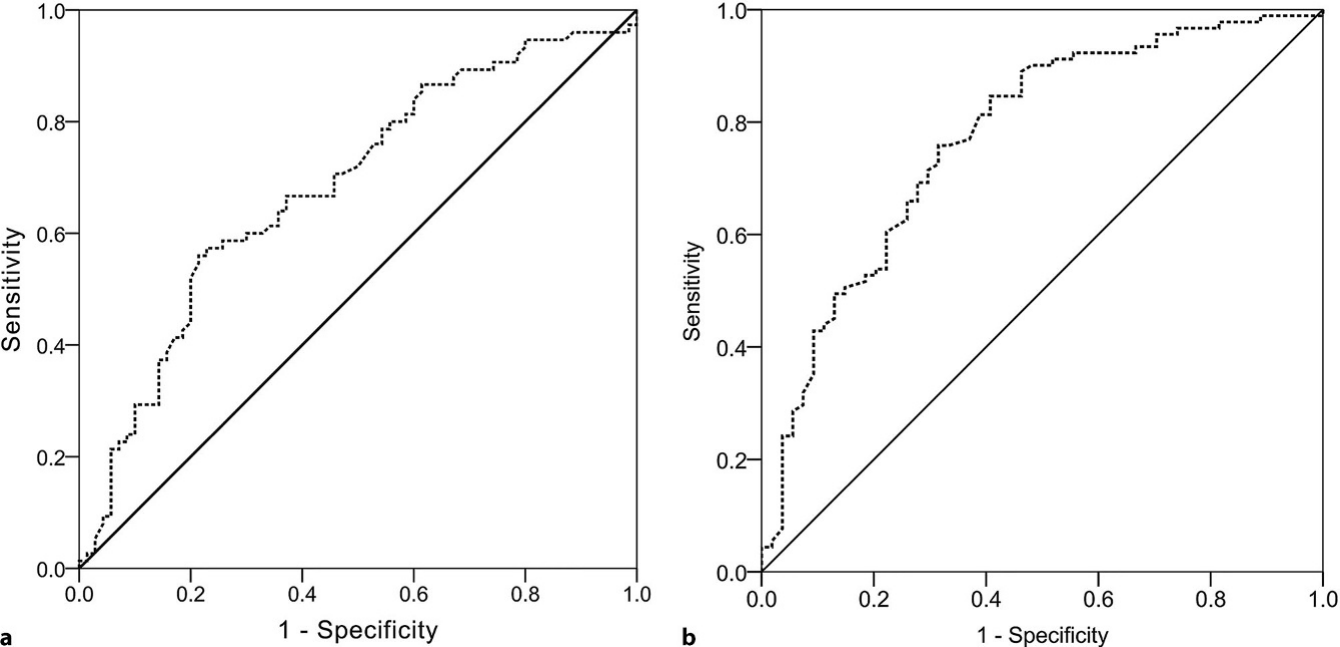
ROC analysis of serum albumin and 90-day recurrence (**a**) and 1‑year all-cause mortality (**b**). Serum albumin <33.1g/l was predictive for 90-day recurrence with a sensitivity of 56% and a specificity of 80% (Youden index 1.35, AUC 0.683), while serum albumin <29.2g/l was predictive for 1‑year all-cause mortality with a sensitivity of 75% and a specificity of 70% (Youden index 1.44, AUC 0.763)

The ATLAS score was unable to predict 90-day recurrence (*p* = 0.064, hazard ratio, HR 1.134, [0.993;1.295]). Also, after exclusion of the ATLAS groups 8–12, ATLAS was unable to predict 90-day recurrence (*p* = 0.103; HR 1.136, [0.975;1.325]). Of all individual ATLAS variables, only serum albumin was predictive of 90-day recurrence (*p* = 0.016, HR 0.958 [0.926;0.992]; Table 4). At ROC analysis the highest Youden index for serum albumin was at a threshold of 33.1g/l. Serum albumin <33.1g/l was predictive for 90-day recurrence with a sensitivity of 56% and a specificity of 80% (Youden index 1.35, area under the curve, AUC 0.683; Fig. 1a).

**Table 4:** Univariate analysis of ATLAS and each of its variables with 90-day recurrence, disease complications, and mortality

	90-day recurrence^a^ *p*-value (HR [95% CI])	Disease complications *p*-value (HR [95% CI])	Mortality^b^ *p*-value (HR [95% CI])
*ATLAS, per point*	0.064 (1.134 [0.993;1.295])	^**c**^**, <0.001** (1.547 [1.266;1.889])	**<0.001** (1.374 [1.194;1.583])
*Age, per 1 year*	0.382 (1.006 [0.992;1.021])	**0.003 **(1.030 [1.010;1.051])	**0.012** (1.021 [1.005;1.038])
*Temperature, per °C*	0.364 (1.202 [0.820;1.764])	0.108 (1.529 [0.911;2.567])	0.308 (1.223 [0.830;1.802])
*Leukocyte count, per G/l*	0.242 (1.025 [0.984;1.068])	0.521 (1.019 [0.962;1.079])	**0.001** (1.061 [1.025;1.099])
*Albumin, per g/l*	**0.016** (0.958 [0.926;0.992])	**<0.001** (0.865 [0.809;0.924])	**<0.001** (0.923 [0.896;0.950])
*Systemic antibiotics*	0.117 (1.677 [0.879;3.197])	**0.007** (3.105 [1.361;7.086])	0.075 (1.813 [0.942;3.490])
*Serum creatinine, per mg/dl*	0.868 (0.983 [0.807;1.199])	0.250 (1.146 [0.908;1.447])	0.324 (1.077 [0.929;1.250])

^a^*p*-values hazard ratios (HR) and confidence intervals (CI) derived from logistic regression analysis of time to recurrence

^b^*p*-values hazard ratios (HR) and confidence intervals (CI) derived from Cox regression analysis

^c^Somers’ D was calculated for the ordinal variable ATLAS, per point (*D* = 0.382)

Bold values represent *p*‑values < 0.05.

### 1-year all-cause mortality

**Fig. 2:**
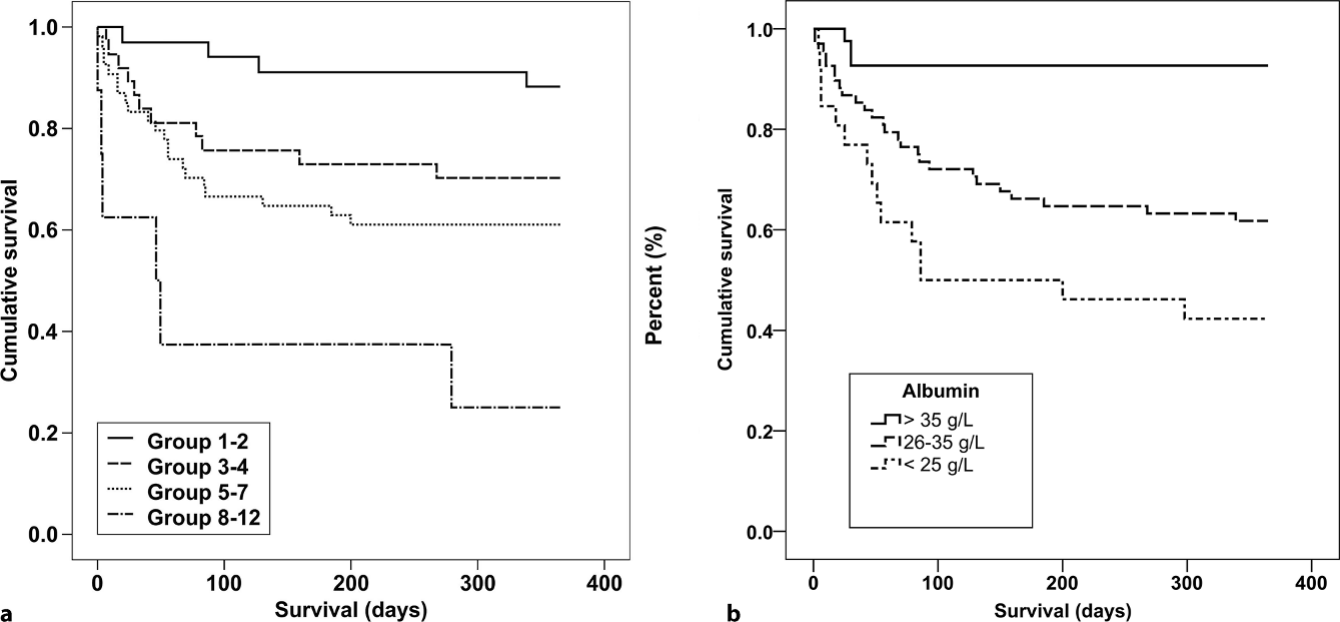
Kaplan-Meier estimates of cumulative survival by ATLAS groups (logrank test *p* < 0.001) (**a**) and serum albumin (logrank test *p* < 0.001) (**b**)

Survival data were available for 135 patients. Of these 44 patients (30.6%) died within 1 year after diagnosis of CDI, of which 34 died within 90 days; however, 14 of these patients experienced recurrence before death. The ATLAS score was strongly associated with 1‑year all-cause mortality (*p* < 0.001, HR by one point increase 1.374 [1.194;1.583]; Fig. 2a). In addition, association between the ATLAS variables age, leukocyte count, and albumin was statistically significant (Table 4). In Fig. 2 Kaplan-Meier estimates of cumulative survival stratified by ATLAS groups and serum albumin are depicted. At ROC analysis the highest Youden index for serum albumin was at a threshold of 29.2g/l. Serum albumin <29.2g/l was predictive for 1‑year all-cause mortality with a sensitivity of 75% and a specificity of 70% (Youden index 1.44, AUC 0.763; Fig. 1b).

### Disease complications

A total of 71 patients (49.3%) experienced no complications, 24 (16.7%) had mild and 49 (34.0%) severe complications while suffering from CDI (Table 5). The rate of severe complications increased with increasing ATLAS score from 6.7% of patients in ATLAS groups 1–2 to 57.1% of patients in ATLAS groups 8–12 (Table 5). Higher ATLAS values were significantly associated with disease complications (*D* = 0.382, *p* < 0.001, HR 1.547 [1.266;1.889]).

**Table 5:** Stratification of disease complications by ATLAS group and serum albumin levels

		Complications			
		None (1)	Mild (2)	Severe (3)	Total
*ATLAS*	*Group 1–2, n (%)*	25 (69.4)	5 (13.9)	6 (16.7)	36
	*Group 3–4, n (%)*	234 (51.1)	7 (15.6)	15 (33.3)	45
	*Group 5–7, n (%)*	21 (37.5)	11 (19.6)	24 (42.9)	56
	*Group 8–12, n (%)*	2 (28.6)	1 (14.3)	4 (57.1)	7
	*–*	71	24	49	144
*Albumin*	*>35* *g/l*	37 (77.1)	4 (8.3)	7 (14.6)	48
	*26–35* *g/l*	27 (40.3)	14 (20.9)	26 (38.8)	67
	*<25* *g/l*	7 (24.1)	6 (20.7)	16 (55.2)	29
	**–**	71	24	49	144

Percentages in parentheses are row percentages

Regarding the individual ATLAS variables, higher age (*p* = 0.003, HR 1.030 [1.010;1.051]), low serum albumin (*p* < 0.001, HR 0.865 [0.809;0.924]), and systemic antibiotic treatment (*p* = 0.007, HR 3.105 [1.361;7.086]) showed a strong association with the occurrence of disease complications, while body temperature (*p* = 0.108, HR 1.529 [0.911;2.567]), white blood count (*p* = 0.521, HR 1.019 [0.962;1.079]) and serum creatinine (*p* = 0.250, HR 1.146 [0.908;1.447]) were not associated with disease complications (Table 4).

## Discussion

Novel drugs have been shown to reduce recurrent infections with *C. difficile* [3, 6–9, 11–13]; however, these drugs are expensive and should therefore only be administered to a selected patient group at high risk for CDI recurrence [17]. Early prevention of disease recurrence is crucial to decrease mortality, which has been shown in several studies (*p* < 0.001) [26]. Considering the data in this study, the ATLAS score was unable to predict recurrence of disease within the first 90 days after onset of CDI, which supports the data published by Jacobson and Slain who showed that a low ATLAS score was associated with higher cure rates of CDI in patients treated with oral metronidazole and/or vancomycin, but was unable to predict CDI recurrence [23]. Suspecting patients in ATLAS group 8–12 to be more likely to experience fatal outcomes before recurrence of disease was even possible to occur, this group was excluded in an additional sensitivity analysis of 90-day recurrence. Nevertheless, no significant association between ATLAS and 90-day recurrence could be demonstrated (*p* = 0.064, HR 1.134 [0.993;1.295]).

The ATLAS score has been shown to be a suitable tool for the prediction of disease complications (*D* = 0.382, *p* < 0.001, HR 1.547 [1.266;1.889]) and mortality (*p* < 0.001, HR 1.374 [1.194;1.583]; Table 4) [22, 24]; however, this study suggests that ATLAS cannot predict 90-day recurrence and therefore is not an adequate tool for the early identification of patients at high risk of CDI recurrence.

The risk factors included in the MODIFY statistical analysis plan have been shown to be appropriate to identify patients at high risk of CDI [10, 12]. The MODIFY plan included the parameters age ≥65 years, history of CDI, compromised immunity, severe CDI and ribotypes 027/078/244, of which all have been shown to increase the risk for recurrent CDI and related adverse outcomes [12, 27–35]. In contrast to previously published data, this study was not able to show an association between age and recurrence of disease in the study population (*p* = 0.382, HR 1.006 [0.992;1.021]) [27, 28]. History of CDI, which served as prognostic marker in the MODIFY trials, was an exclusion criterion in this study. This study utilized severity (i. e. disease complications) of CDI as outcome parameter rather than as predictor for recurrence of disease. The ATLAS score, for example, was strongly associated with disease complications (*D* = 0.382, *p* < 0.001, HR 1.547 [1.266;1.889]), but not with recurrence of disease (*p* = 0.064, HR 1.134 [0.993;1.295]). Nevertheless, disease severity can predict further progression and recurrence of disease but is usually not known at the time diagnosis of CDI is established. The ribotypes 027/078/244 have been shown to be types with increased virulence; however, it is likely that further lineages may evolve due to the dynamic development of the species.

The simple and cheap to measure continuous numerical laboratory parameter, serum albumin was the only assessed parameter able to predict disease complications (*p* < 0.001, HR 0.865[0.809;0.924]), mortality (*p* < 0.001, HR 0.923 [0.896;0.950]) and recurrence of disease (*p* = 0.016, HR 0.958 [0.926;0.992]). This can be explained by several mechanisms, including serum albumin’s function as a negative acute phase protein, which negatively correlates with tissue inflammation and thereby adverse outcomes [36]. Furthermore, CDI is known to be a protein loss enteropathy. Severe infection even presents with intestinal pseudomembranes consisting of protein due to increased intestinal permeability, giving it the name pseudomembranous colitis [36, 37]. This is another reason likely to explain serum albumin’s close association with disease complications, recurrence of disease and mortality. Furthermore, it is important to mention that all variables included in analyses, including serum albumin, were taken from the first day patients presented with diarrhea. In a retrospective case-control study published by Rotramel et al. mean admission albumin levels were not able to predict 60-day recurrence, while mean nadir albumin levels were [38]; however, it is unclear whether the admission albumin level was actually determined at the time of diagnosis. In contrast, in this study serum albumin levels at onset of diarrhea were able to predict 90-day recurrence of CDI. In ROC analysis serum albumin at a threshold of <33.1g/l was identified to be predictive for 90-day recurrence (AUC 0.683, sensitivity = 56%, specificity of 80%) and serum albumin <29.2g/l to be predictive for 1‑year all-cause mortality (AUC 0.763, sensitivity = 75%, specificity = 70%).

In conclusion, despite the ATLAS score’s good performance in prediction of disease complications and mortality, decisions on the application of novel treatment cannot be made based on this score, as it does not correlate with 90-day recurrence of CDI; however, a decrease in serum albumin was highly correlated with 90-day recurrence of disease.

Besides the proposed parameters by the MODIFY study plan, which have been shown to indicate a high risk for recurrent CDI, monitoring serum albumin levels during acute infection with *C. difficile *is suggested. With a relatively high prevalence of recurrence of approximately 30% in this study, the positive predictive value is 55%, which is too low to guide decisions about medication; however, the negative predictive value under these circumstances is 81% and increases with decreasing prevalence. Therefore, standard therapy should be applied as long as serum albumin does not decline below 33 g/l. Below this threshold, it is a matter of the individual judgment taking all other conditions of the patient into consideration. Further studies are needed to evaluate which serum albumin threshold justifies the application of novel therapies to reduce recurrence of disease. To our knowledge this is the first study showing an association of serum albumin at onset of diarrhea with 90-day recurrence of CDI.
